# Public Interest in Neurological Diseases on Wikipedia during Coronavirus Disease (COVID-19) Pandemic

**DOI:** 10.3390/neurolint13010006

**Published:** 2021-02-10

**Authors:** Stela Rutovic, Ana Isabel Fumagalli, Inna Lutsenko, Francesco Corea

**Affiliations:** 1 Department of Neurology, University Hospital Dubrava, Avenija Gojka Suska 6, 10000 Zagreb, Croatia; 2 Department of Neurology, Sanatorio Parque, Rosario S2000, Argentina; anisaf7@yahoo.com; 3 Center for Distance Learning and Advanced Training, Kyrgyz State Medical Academy after I.K.Akhunbaev, Bishkek 720020, Kyrgyzstan; ilutsenko555@gmail.com; 4 Stroke and Neurology Unit, San Giovanni Battista Hospital, 06034 Foligno, Italy; fcorea@gmail.com

**Keywords:** Wikipedia, COVID-19, neurological diseases

## Abstract

Infodemiology is a research discipline that investigates parameters of information distribution in order to support public health and public policy. Wikipedia, a free online encyclopedia, is commonly used as a source of information for infodemiological studies. Using Pageviews analysis, we descriptively assessed the total monthly number of views of the Wikipedia articles in English describing main neurological diseases in the period from January 2018 to July 2020. Our results show a general trend of a decrease in interest in neurological disease-related pages throughout years and especially during the burst of interest towards coronavirus. The monitoring of infodemiological indicators shall be prioritized to reshape global campaigns and tailored advocacy programs.

## 1. Introduction

Eysenbach defined infodemiology as a concept related to “the science of distribution and determinants of information in an electronic medium, specifically the Internet, or in a population, with the ultimate aim to inform public health and public policy”. Some of the most popular web services used for infodemiological studies are Google trends which provide data about volume of Google search on specific topics [1,2] and Pageviews analysis of Wikipedia which is the most frequently used website for medical information by both healthcare professionals and patients [3].

From December 2019, the performance of many healthcare systems has been severely challenged by coronavirus disease (COVID-19) outbreaks. The global interest of the authorities as well as of the general public was immediately focused on the pandemic. A negative impact on the treatment of neurological diseases was observed [4]. The aim of our study was to descriptively analyze the total monthly number of views of the Wikipedia articles in English on the main neurological diseases during the coronavirus pandemic compared to previous years.

## 2. Materials and Methods

Using Pageviews analysis [5] we assessed the total monthly number of views of the English Wikipedia articles devoted to main neurological diseases and coronavirus from January 2018 to July 2020.

Pageviews analysis is a free online tool which provides total pageviews statistics across all Wikipedia sites and enables comparison of views across different Wikipedia articles.

The total monthly number of views of Wikipedia articles on the following diseases was assessed for the period from January 2018 to July 2020: stroke, multiple sclerosis (MS), epilepsy, Alzheimer’s disease (AD), Parkinson’s disease (PD), and coronavirus.

The total monthly number of views in the period from February to March 2019 was compared to total number of views in the period from February to March 2020.

## 3. Results

**Figure 1:**
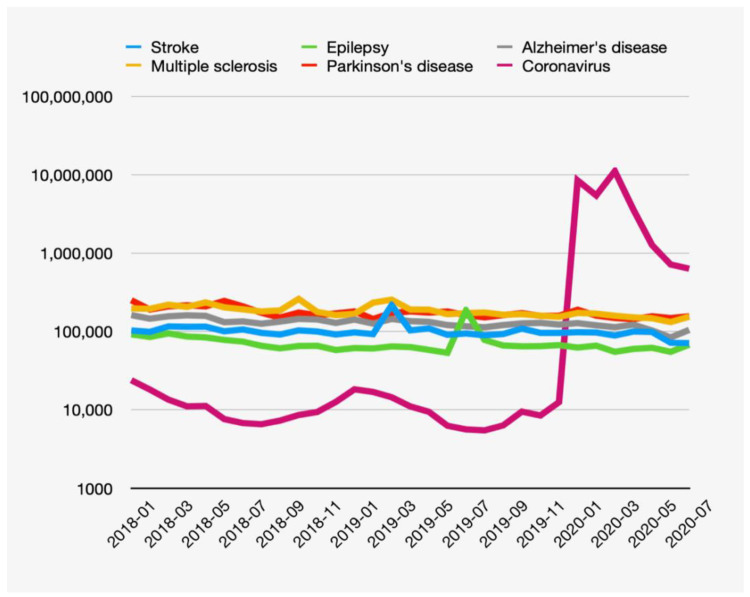
Trends in monthly views of Wikipedia pages for selected neurological conditions and coronavirus from January 2018 to July 2020 presented in a logarithmic scale.

The number of views per month of the articles on selected neurological diseases ranged from 53,000 to 253,000 per month (Figure 1). The neurological disease with the most views was MS (range of views from 131,000 in June 2020 to 256,000 in March 2019 (mean value= 183,836). The number of monthly views of the stroke articles ranged from 70,877 to 215,656 (mean value= 103,954), for epilepsy from 53,249 to 184,440 (mean value= 74,337), for AD from 83,780 to 161,517 (mean value= 129,266), and for PD it ranged from 143,319 to 249,606 (mean value= 176,881) views.

Interest in Wikipedia articles on coronavirus ranged from 5400 to 23,000 views per month before December 2019. After the onset of coronavirus pandemic, the monthly number of views of the coronavirus articles reached 11,000,000.

According to our results, there is a general trend of a decrease in interest in neurological diseases related to Wikipedia pages from January 2018 to July 2020, with increased interest in the articles on coronavirus from January to March 2020. The total decrease in monthly views (February/March 2020 compared to the same period 2019) for all selected neurological diseases was 21.9%, ranging from 2.5 to 39% The most pronounced decrease in views was for stroke (39%), followed by MS (32%), and the lowest was for PD (2.5%) (Table 1).

Isolated peaks of interest in stroke (215,656 views in March 2019) and epilepsy (184,440 views in July 2019) were observed.

## 4. Discussion

The results of our descriptive data analysis showed a decreased interest in English Wikipedia articles on selected neurological diseases throughout years, with several peaks of increased interest. The decrease in interest was especially pronounced in the weeks before March 2020, when the World Health Organization (WHO) announced the COVID-19 pandemic [6]. At that time, the exponential increase in interest for articles on coronavirus was observed.

When comparing total views of Wikipedia articles on selected neurological diseases in February and in March 2019 with the same time period in 2020, there is a total decrease in interest in selected neurological diseases during the coronavirus pandemic of 21.9%, ranging from 2.5 to 39%. The biggest decrease was observed for stroke, and the smallest for PD. There was a peak of interest in stroke in March 2019 which correlates with the incident of actor Luke Perry suffering a stroke. Another peak of views is visible for the article on epilepsy in July 2019. This may be easily associated with the sudden death of another celebrity: Cameron Boyce. This finding is consistent with previous studies which suggested that peaks in search volumes of Wikipedia articles were temporally related to news about famous people suffering from neurological disorders, or “breaking news” or mass-media events referring to the neurological diseases [3].

Some studies have shown that online interest in borreliosis and in West Nile virus disease correlates with their prevalence [7]. Most studies of neurological diseases did not show a correlation of web visitors’ interest with the disease prevalence [3,8]. A study investigating Wikipedia visits related to the most common neurological diseases (MS, AD, stroke, epilepsy) did not find any relation between the incidence or prevalence of neurological disorders and the search volume for the related Wikipedia articles. They found that the interest in MS on Wikipedia was much higher than for migraine, epilepsy and stroke. This finding can be explained in a way that the MS patients are young Internet users, contrary to the older people who suffer from stroke or PD more frequently. Additionally, an increasing number of incidental imaging findings (such as white matter lesions) obtained during diagnostic workup for a wide range of neurological symptoms, results in increased interest in Wikipedia articles [3].

Several other studies showed that interest in MS on Wikipedia was higher than the interest in other neurological diseases [8,9].

A study of searches for epilepsy- and seizures-related Wikipedia articles also found that the Wikipedia pages on MS were much more frequently visited than the pages on epilepsy, despite the fact that MS is a less common neurological disorder. The authors found that the Wikipedia articles on syncope were much less often visited than the articles on epilepsy, although seizures and epilepsy are much rarer events than syncope. The authors suggest that the high number of Google and Wikipedia searches related to epileptic events reflects patients’ fears and worries for seizures, which are commonly perceived as more serious events than syncope [9].

A study investigating the seasonality of Google Trends searches on MS and on MS-related terms in the Southern and Northern hemispheres showed peaks of interest in spring and autumn, possibly as a result of seasonal variations in the disease burden [10]. Therefore, it is possible that the reduced interest in neurology-related Wikipedia web pages might have corresponded to a reduced interest in respective neurological diseases.

During the COVID-19 pandemic, the number of hospital admissions for stroke patients has declined. This phenomenon can be explained by patients’ fear of COVID-19 infection as well as due to social distancing measures. Stroke patients usually have more comorbidities, which present a risk factor for adverse COVID-19 infection outcomes. Therefore, patients with mild clinical presentation may avoid hospitalizations, leading to increased number of hospital admissions with severe stroke [4,11,12]. The pandemic also accentuated healthcare disparities for neurological patients, negatively affecting socially disadvantaged groups. Patients with lower socioeconomic status, ethnic and racial minorities had reduced access to hospital care and worse outcomes [13].

Decrease in interest for other medical conditions and procedures during the coronavirus pandemic, such as rheumatic diseases, otholaryngology and clinical imaging, has been observed [14,15,16].

A correlation has been found between online interest in COVID-19 and reported cases as well as deaths. In the same study, a critical point, after which the relationship strength decreases were identified, suggested that an infodemiological approach can be efficient in regions or countries that have not yet peaked in terms of COVID-19 cases [17].

We can assume that the increased internet interest for COVID-19-related articles reflects fears of an unknown and potentially severe or life-threatening disease. Influential celebrities with a strong web presence suffering from COVID-19 have also shared their experience with the disease which has contributed to the increased interest.

The main limitations of our study are not including quantitative analyses of temporal variations of search trends, usage of English terms only, as well as a lack of information on who actually uses Wikipedia, i.e., the patients, relatives or physicians.

As far as we know, this is the first infodemiological study investigating the potential effects of the COVID-19 on public interest in neurological diseases.

According to our results, the current COVID-19 pandemic had relevant short-term negative effects on the public interest in neurological diseases. Monitoring of infodemiological parameters can be used to reshape global health campaigns and to tailor advocacy programs. This was also suggested in a recent WHO framework for managing an infodemic in health emergencies [18,19].

## Figures and Tables

**Table 1:** Total views of the English Wikipedia articles on each neurological disease in February and March 2019 and the same time period in 2020.

Neurological Disease	Views Feb–Mar 2019	Views Feb–Mar 2020	Difference in Percentage
Stroke	308,075	185,670	−39
Epilepsy	124,510	120,226	−3.4
Multiple Sclerosis	486,386	327,178	−32
Alzheimer’s disease	269,938	232,947	−13
Parkinson’s disease	315,568	3,076,011	−2.5
Total	1,504,477	1,173,622	−21.9

## Data Availability

Data available at: https://pageviews.toolforge.org/?project=en.wikipedia.org&platform=all-access&agent=user&redirects=0&start=2018-01&end=2020-07&pages=Stroke|Epilepsy|Alzheimer%27s_disease|Multiple_sclerosis|Parkinson%27s_disease|Coronavirus (accessed on 17 December 2020).
